# Supplementary material for: Impaired Butyrate Induced Regulation of T Cell Surface Expression of CTLA-4 in Patients with Ulcerative Colitis
Source: Int J Mol Sci. 2021 Mar 17;22(6):3084. doi: 10.3390/ijms22063084 (PMC8002718; doi:10.3390/ijms22063084)
Supplement: Supplementary file 1 [file ijms-22-03084-s001.zip › Supplementary material.docx]

# Impaired butyrate induced regulation of T cell surface expression of CTLA-4 in patients with ulcerative colitis

Maria K. Magnusson^1^, Alexander Vidal^2^, Lujain Maasfeh^1^, Stefan Isaksson^1^, Rajnees Malhotra^3^, Henric K. Olsson^3,†^ and Lena Öhman^1,†,^*.

^1^Department of Microbiology and Immunology, Institute of Biomedicine, Sahlgrenska Academy, University of Gothenburg, Gothenburg, Sweden; [maria.magnusson@microbio.gu.se](mailto:maria.magnusson@microbio.gu.se) (M.K.M); [lujainmaasfeh@gmail.com](mailto:lujainmaasfeh@gmail.com) (L.M); [stefan.isaksson@microbio.gu.se](mailto:stefan.isaksson@microbio.gu.se) (S.I); [lena.ohman@gu.se](mailto:lena.ohman@gu.se) (L.Ö)

^2^Bioscience In Vivo, Research and Early Development, Respiratory & Immunology, BioPharmaceuticals R&D, AstraZeneca, Gothenburg, Sweden; [alexander.vidal@astrazeneca.com](mailto:alexander.vidal@astrazeneca.com)

^3^Translational Science and Experimental Medicine, Research and Early Development, Respiratory & Immunology, BioPharmaceuticals R&D, AstraZeneca, Gothenburg, Sweden; [rajneesh.malhotra@astrazeneca.com](mailto:rajneesh.malhotra@astrazeneca.com); [henric.k.olsson@astrazeneca.com](mailto:henric.k.olsson@astrazeneca.com)

^†^These authors share last authorship.

*Correspondence: [lena.ohman@gu.se](mailto:lena.ohman@gu.se)

Supporting information.


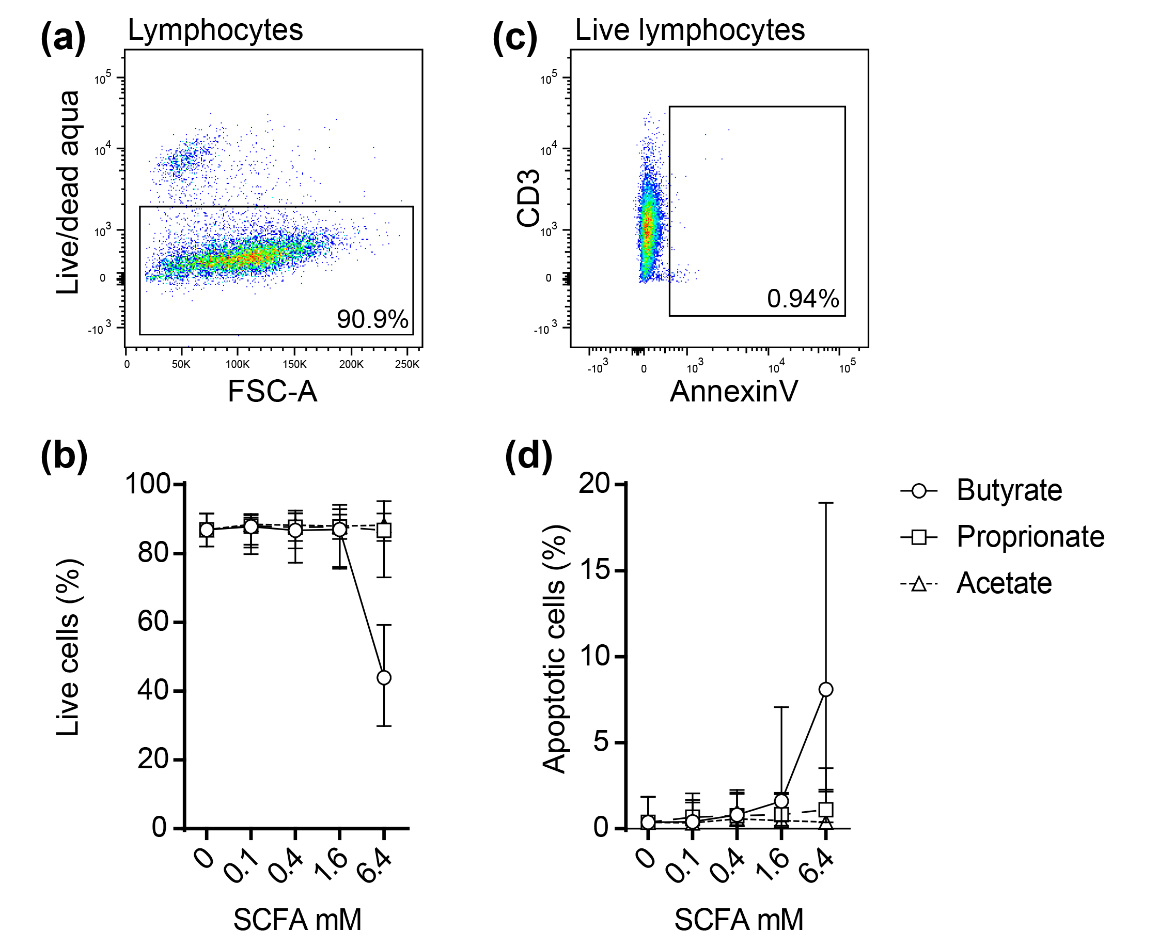


**Figure S1.** Effects of SCFAs on cell viability and apoptosis. PBMCs from healthy subjects were stimulated with anti-CD3 and anti-CD28 with increasing concentrations of butyrate, proprionate or acetate (0-6.4 mM) for 72h and analyzed by flow cytometry. **(a)** Live lymphocytes were defined as live/dead aqua negative cells (or 7AAD negative cells). **(b)** Apoptotic cells were defined as AnnexinV^+^​ cells among live lymphocytes. The frequencies of live lymphocytes **(c)** and apoptotic cells **(d)** are shown (n=9). Data are shown as median IQR.

**
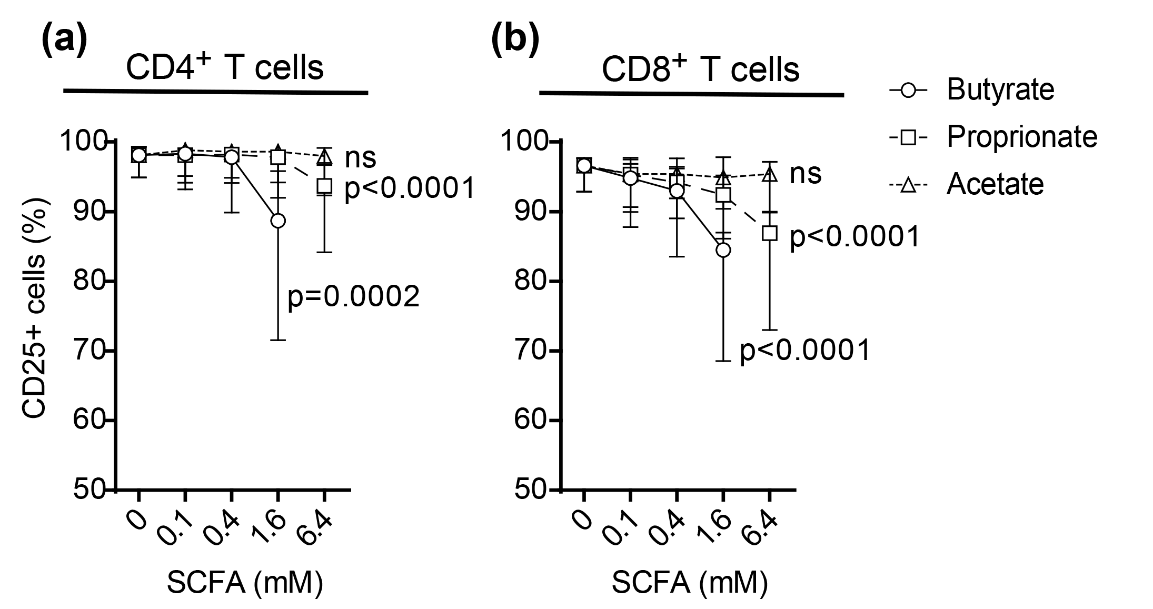
**

**Figure S2.** Effects of butyrate and proprionate on the frequencies of activated T cells. PBMCs were stimulated with anti-CD3 and anti-CD28 with increasing concentrations of butyrate (0-1.6 mM), proprionate (0-6.4 mM) or acetate (0-6.4 mM) for 72h and analyzed by flow cytometry. The frequencies of activated CD4​^+^ **(a)** and CD8^+^​ **(b)** T cells from healthy subjects are shown (n=9). Data are shown as median IQR.

**
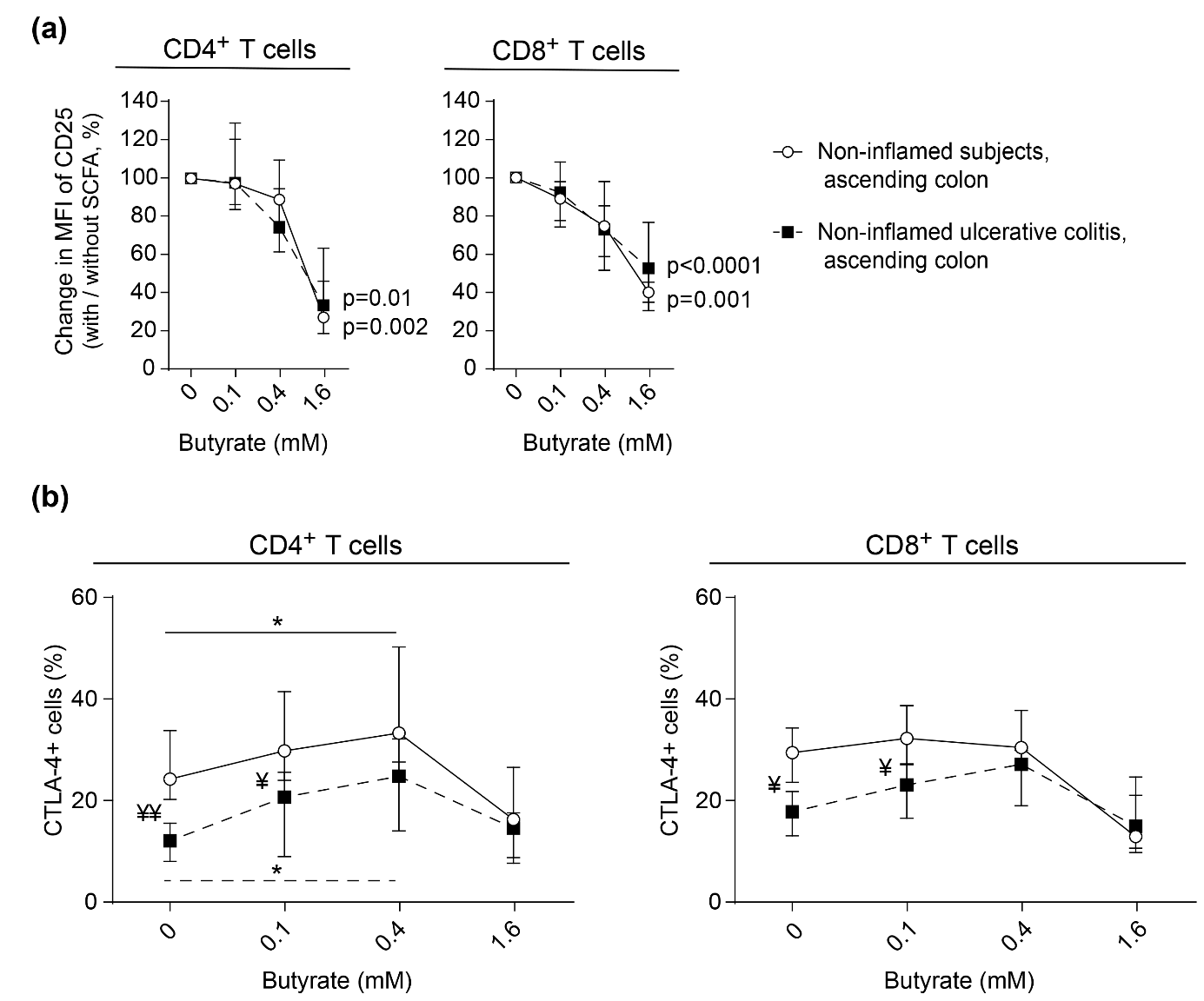
**

**Figure S3.** Effects of butyrate on activation and CTLA-4 expression on T cells from ascending colon. Lamina​ propria (LP) cells were stimulated with anti-CD3 and anti-CD28 with increasing concentrations of butyrate (0-1.6 mM) for 48h and analyzed by flow cytometry. **(a)** Median fluorescent intensity (MFI) of CD25 and **(b)** frequency of CTLA-4 expression in stimulated CD4^+^​ (left) and CD8^+^​ (right) T cells from non-inflamed ascending colon of UC patients (n=6) and non-inflamed sigmoid colon of subjects (n=6). Data are shown as median IQR.
