# Supplementary figures and images for: Impaired Butyrate Induced Regulation of T Cell Surface Expression of CTLA-4 in Patients with Ulcerative Colitis
Source: Int J Mol Sci. 2021 Mar 17;22(6):3084. doi: 10.3390/ijms22063084 (PMC8002718; doi:10.3390/ijms22063084)

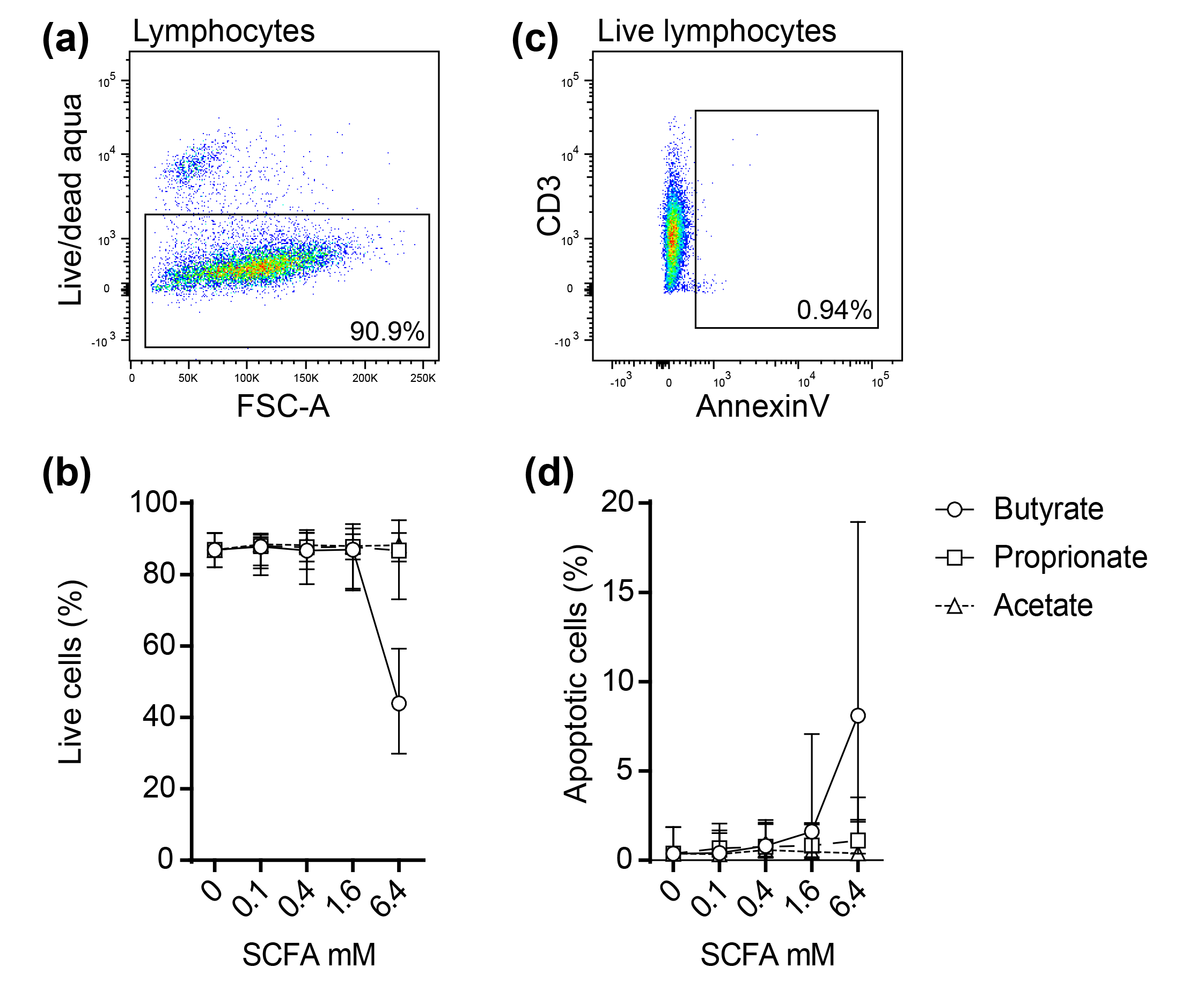

Supplement: Supplementary file 1 [file ijms-22-03084-s001.zip › Supplementary figure 1.tif]

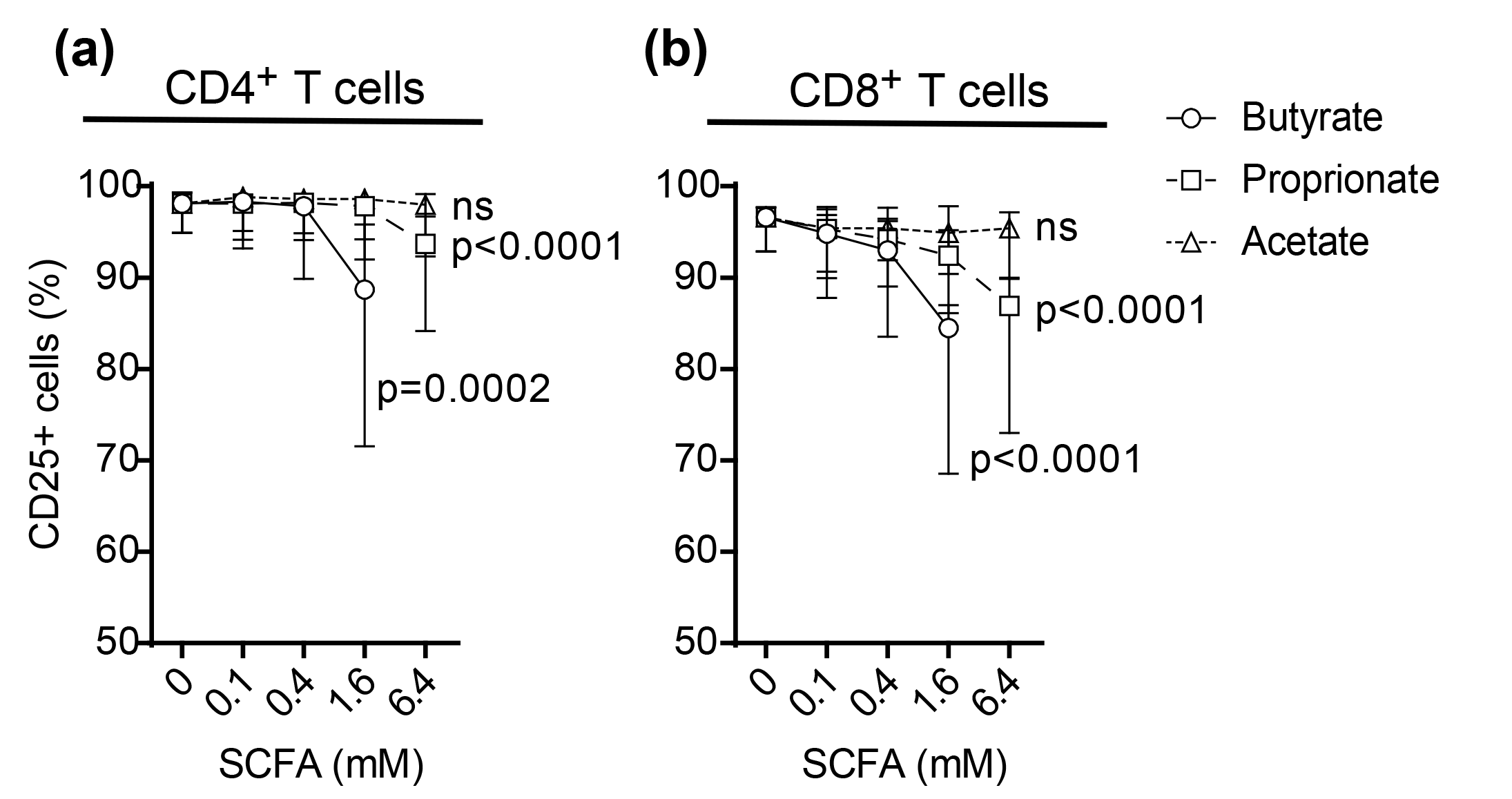

Supplement: Supplementary file 1 [file ijms-22-03084-s001.zip › Supplementary figure 2.tif]

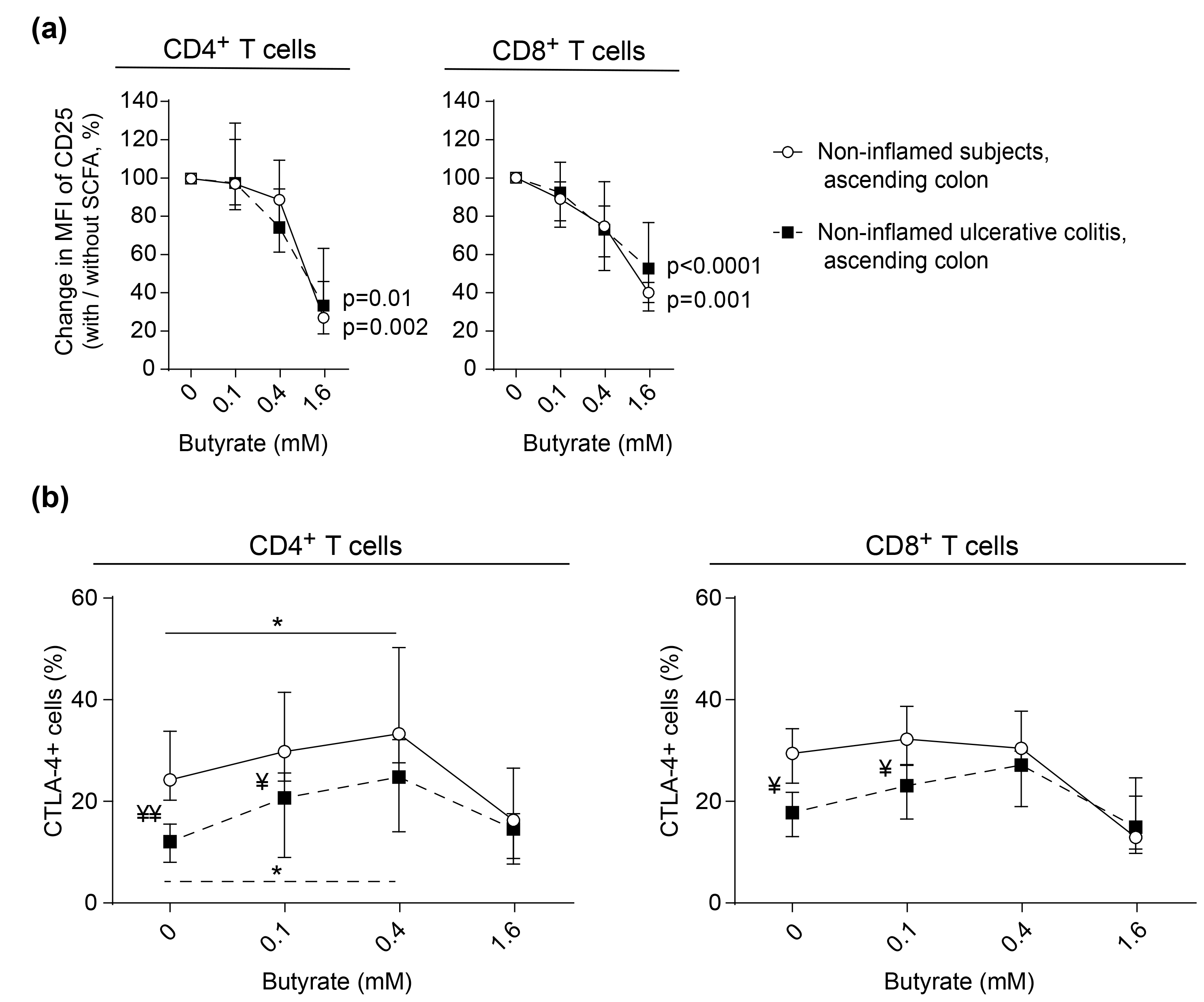

Supplement: Supplementary file 1 [file ijms-22-03084-s001.zip › Supplementary figure 3.tif]
